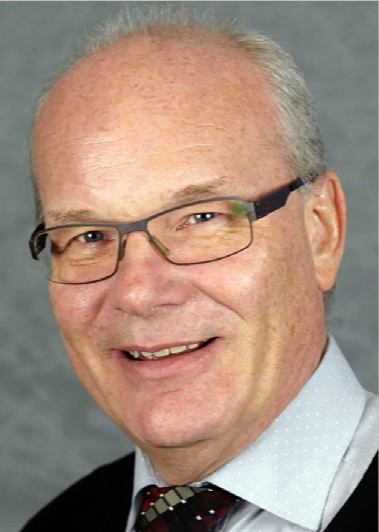# Small‐Molecule Inhibition of Glucose Transporters GLUT‐1–4

**DOI:** 10.1002/cbic.201900544

**Published:** 2019-11-08

**Authors:** Elena S. Reckzeh, Herbert Waldmann

**Affiliations:** ^1^ Department Chemical Biology Max Planck Institute of Molecular Physiology Otto-Hahn-Strasse 11 44227 Dortmund Germany; ^2^ Department Chemistry and Chemical Biology TU Dortmund University Otto-Hahn-Strasse 4a 44227 Dortmund Germany

**Keywords:** antitumor agents, cancer, drug discovery, GLUT inhibitors, metabolism

## Abstract

Glucose addiction is observed in cancer and other diseases that are associated with hyperproliferation. The development of compounds that restrict glucose supply and decrease glycolysis has great potential for the development of new therapeutic approaches. Addressing facilitative glucose transporters (GLUTs), which are often upregulated in glucose‐dependent cells, is therefore of particular interest. This article reviews a selection of potent, isoform‐selective GLUT inhibitors and their biological characterization. Potential therapeutic applications of GLUT inhibitors in oncology and other diseases that are linked to glucose addiction are discussed.

## Graphical abstract

**Targeting aerobic glycolysis** by means of glucose uptake inhibition may be a new approach for the treatment of proliferative diseases such as cancer and inflammatory disorders. This Minireview provides an overview of the most potent and advanced inhibitors of glucose uptake by glucose transporters GLUT‐1–4 and sheds light on their potential for therapeutic applications.

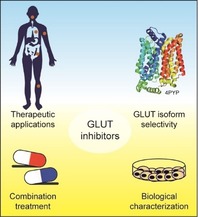

## 1 Introduction

**Figure 1:**
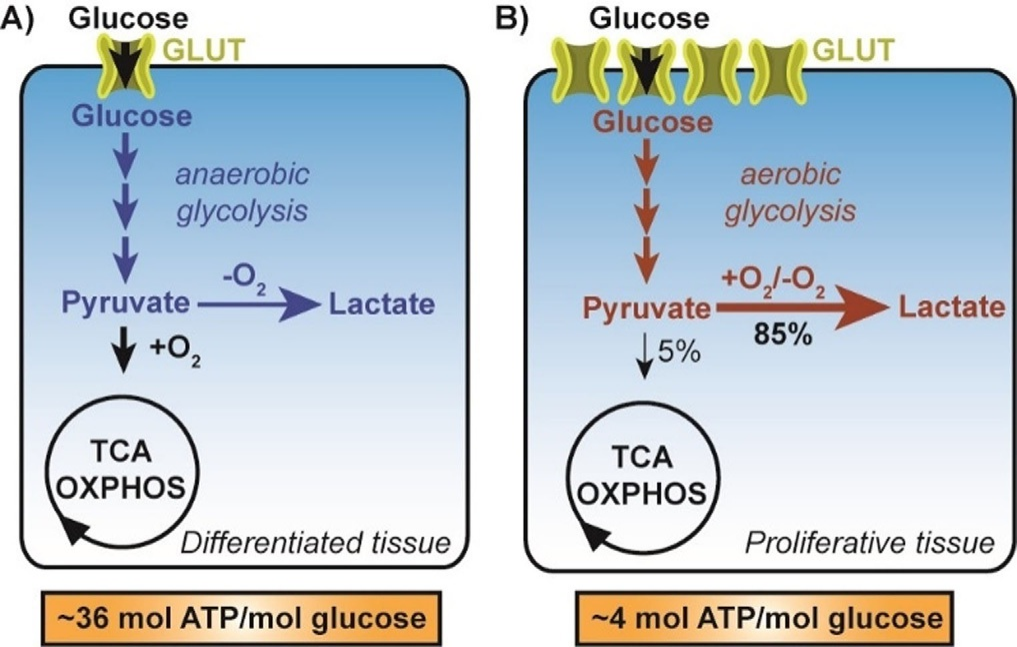
Schematic representation of the major metabolic pathways in A) differentiated and B) proliferative tissues. TCA: tricarboxylic acid cycle; OXPHOS: oxidative phosphorylation; GLUT: facilitative glucose transporter.

Hyperproliferation is widely associated with dysregulated energy metabolism in order to fuel growth and cytokinesis. Thereby it is linked to various diseases such as cancer,^1^ autoimmune diseases,^2^ and fibrosis.^3^ The altered energy metabolism of cancer was first investigated by Otto Warburg in 1924.^4^ He observed that cancer cells perform glycolysis and ferment the thereby generated pyruvate to lactate irrespective of oxygen availability (Figure 1 B). This phenomenon was termed aerobic glycolysis, or the Warburg effect, and yields about 4 mol ATP per mol glucose.^1^ Nonmalignant cells fuel pyruvate into the tricarboxylic acid (TCA) cycle and oxidative phosphorylation (OXPHOS) in the presence of oxygen in order to generate approximately 36 mol ATP per mol absorbed glucose (Figure 1 A). The reason for this adapted metabolism remains a matter of debate (e.g., see Liberti and Locasale^5^).

A high rate of glycolysis in cancer depends on key glycolytic enzymes and proteins, especially the glucose transporters GLUT‐1/GLUT‐3, hexokinase 2 and pyruvate kinase 2.^6^ The increased absorbance of glucose by overexpression of GLUTs contributed to the development of positron emission tomography using ^18^F‐labeled 2‐deoxy‐d‐glucose, which is used to visualize tumors within the patient's body.^7^

Targeting the first rate‐limiting step of glycolysis promises to be an effective strategy to limit glucose supply. To date, 14 different GLUT isoforms are known that are subdivided into three distinct protein classes according to their phylogenic homology (e.g., see Barron et al.^8^). Each GLUT isoform has a unique tissue distribution and substrate specificity and fulfills a specific physiological function. The GLUT isoforms GLUT‐1 to ‐4 (class I) were investigated most intensively, with a particular focus on GLUT‐1.^9^

However, no GLUT inhibitor has been advanced to clinical studies.^10^ This review gives an overview of the most promising GLUT inhibitors that were developed within the last 20 years with a view to the treatment of cancer and other disorders.

## 2 Discovery of the Most Potent GLUT Inhibitors

**Figure 2:**
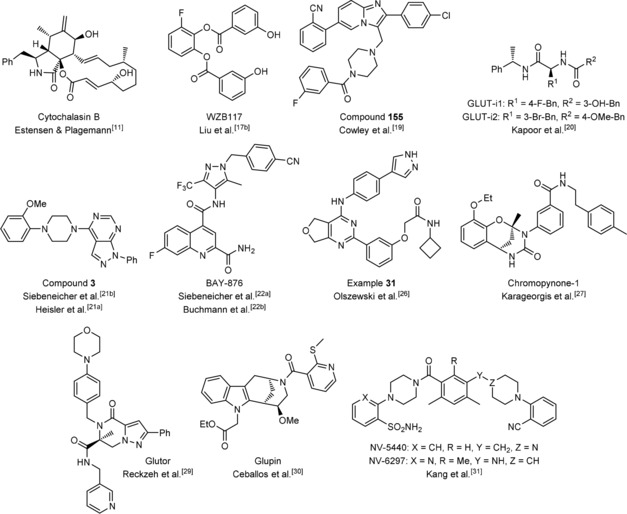
Structures of selected potent GLUT inhibitors.

The fungal metabolite cytochalasin B decreased the glucose supply of cancer cells, which originally spurred the interest in this mode of action.^11^ Cytochalasin B inhibited the uptake of [^14^C]2‐deoxy‐d‐glucose ([^14^C]2DG) in N1S1‐67 cells and its incorporation in lactate with an IC_50_ value below 4 μm in a noncompetitive manner (Figure 2, Tables 1 and 2).^11^, ^12^ In human erythrocytes, cytochalasin B inhibited the uptake of [^14^C]2DG with an IC_50_ value of 0.52 μm.^13^ It targets GLUT‐1 to ‐4 but not GLUT‐7 (Table 2).^14^ Furthermore, cytochalasin B inhibited the growth of murine B16F10 cells with a GI_50_ value of about 0.4 μm as determined after four days of treatment (Table 2).^15^ Although cytochalasin B potently inhibits actin polymerization, which restricts the therapeutic applicability of the natural product, it is often used as a control compound in metabolic studies.^16^ Since the discovery of cytochalasin B, several GLUT inhibitors with varying GLUT isoform selectivity have been described including natural products, non‐natural small molecules and peptide analogues (e.g., see Granchi et al.10b). However, only a few potent compounds (IC_50_<1 μm) were identified (Figure 2, Tables 1 and 2).

**Table 1:** Overview of a selection of highly potent glucose uptake inhibitors.

Name	Class	Assay	Cell line	IC_50_ [nm]
cytochalasin B^11^	natural product	[^14^C]2DG uptake	erythrocytes	520
WZB11717b	small molecule	[^3^H]2DG uptake	A459	500
compound **155** 19	small molecule	[^3^H]2DG uptake	HEK293, hGLUT‐1^[a]^	≈30
GLUT‐i1^20^ GLUT‐i2^20^	peptide analogue	ATP depletion^[b]^	CHO‐K1, hGLUT‐1^[a]^ luciferase^[c]^	267±133 140±72
compound **3** 21b	small molecule	ATP depletion^[b,d]^	DLD‐1	25
BAY‐87622a	small molecule	ATP depletion^[b,d]^	DLD‐1	2
example **31** 26	small molecule	ATP depletion^[d,e]^	HT1080	10–100
chromopynone‐1^27^	small molecule	2DG uptake^[f]^	HCT116	412
glutor^29^	small molecule	2DG uptake^[f]^	HCT116	11
glupin^30^	small molecule	2DG uptake^[f]^	MDA‐MB‐231	4
NV‐5440^31^	small molecule	[^3^H]2DG uptake	MCF7	36

[a] Stable transfection. [b] Rotenone (mitochondrial complex I inhibitor). [c] Constitutive expression. [d] CellTiter‐Glo®. [e] Oligomycin (mitochondrial ATP synthase inhibitor). [f] Resazurin‐coupled.

**Table 2:** Overview of biological data obtained for glucose uptake inhibitors reported in Table 1.

Compound	Selectivity	Mode of action	IC_50_/GI_50_ viability/growth in vitro	Further preclinical data
cytochalasin B^11^	GLUT‐1–414a	non‐competi‐	B16F10 ≈0.4 μm ^[b] [15]^	reduced tumor formation in mice at 10 and
	over 714b	tive^[a] [12b]^		100 mg kg^−1^,^15^ inhibitor of actin polymerization
WZB11717b	GLUT‐4 over	competitive^[a]^	A549 10‐30 μm,^[c]^ NL20 resistant	increased cell sensitivity under hypoxic conditions
	GLUT‐1/312b	12b		in vitro, 70 % tumor volume reduction in xenograft models
compound	n.d.	n.d.	A549 <126 nm (5 mm Glc)^[d]^	–
**155** 19				
GLUT‐i1^20^	GLUT‐1/4	n.d.	n.d.	co‐crystal with hGLUT‐1
GLUT‐i2^20^	over GLUT‐2/3			
compound **3** 21b	GLUT‐1/3	competitive	n.d.	–
	over GLUT‐2			
BAY‐87622a	GLUT‐1 over	competitive	OVCAR‐3 60 nm,^[e] [24]^ BT549, MDA‐MB‐436, HCC70	68 % tumor volume reduction in SKOV‐3
	GLUT‐2/3/4		resistant at 3 μm ^[d] [23]^	xenograft in mice after 2 weeks (4.5 mg kg^−1^)^24^
example **31** 26	n.d.	n.d.	Jurkat 293.5 nm,^[f]^ MOLT‐4 385 nm,^[f]^	–
			U937 256 nm ^[f]^	
chromo‐	GLUT‐1/3	n.d.	HCT116 >25 μm (25 mm Glc), 3.8 μm (5 mm Glc);^[d]^	–
pynone‐1^27^	over GLUT‐2/4		MIA PaCa‐2 2.8 μm (25 mm Glc), 0.6 μm (5 mm Glc)^[d]^	
glutor^29^	GLUT‐1‐3	n.d.	UM‐UC‐3 4 nm, ^[f]^ MIA PaCa‐2 4 nm,^[f]^ PBMCs and	equally potent in 2D and 3D cell culture
	over GLUT‐4		IMR‐90 resistant at 30 μm,^[f]^ 94 cell lines tested^[f]^	
glupin^30^	GLUT‐1/3	mixed‐type	UM‐UC‐3 32 nm,^[f]^ MIA PaCa‐2 61 nm,^[f]^ PBMCs and	metabolomic measurement confirmed reduction
	over GLUT‐2/4	inhibition	IMR‐90 resistant at 30 μm ^[f]^ 94 cell lines tested^[f]^	of glycolytic metabolites in MOLT16 cells
NV‐5440^31^	GLUT‐1–4	n.d.	n.d.	confirmed inhibition of mTORC1 activity in mice
	over GLUT‐5			(30 mg kg^−1^, IP)

n.d.: not determined, Glc: glucose, IP: intraperitoneal injection. [a] Of glucose uptake. [b] Cell counter after 4 days. [c] MTT assay after 48 h. [d] Employing live‐cell imaging. [e] MTT assay after 72 h. [f] Sulforhodamine B assay after 72 h.

The phenolic WZB117 (Figure 2) inhibits uptake of [^3^H]2DG in A549 cells with an IC_50_ value of approximately 500 nm (Table 1) and was found to decrease extracellular lactate levels and the intracellular ATP pool in A549 cells after 6 to 24 hours of treatment.^17^ Proliferation and viability of A549 cells but not of nonmalignant NL20 cells were inhibited by WZB117 with micromolar IC_50_ (Table 2).17b Xenograft studies with A549 cells revealed a decrease in the tumor volume by 70 % after treatment with WZB117 for 70 days (daily intraperitoneal injection, 10 mg kg^−1^). WZB117 inhibits GLUT‐1 as shown via [^3^H]2DG uptake inhibition in red blood cells (which express solely GLUT‐1).17b Although WZB117 has been used as GLUT‐1‐selective inhibitor in multiple studies mostly focusing on cancer,^18^ it was proposed to inhibit mainly GLUT‐4 as determined by GLUT‐1‐, ‐3‐, and ‐4‐overexpressing HEK293 cells ([^3^H]2DG uptake, Table 2).12b Kinetic studies revealed a competitive inhibition of glucose uptake.12b

Researchers at IOmet Pharma described a class of potent imidazopyridine‐derived glucose uptake inhibitors (see, e.g., compound **155**, Figure 2). Compound **155** inhibits the uptake of [^3^H]2DG in HEK293 cells that stably overexpress hGLUT‐1 with an IC_50_ value of approximately 30 nm (Table 1).^19^ The isoform selectivity of 59 derivatives within a collection of 385 compounds determined for GLUT‐1 to ‐4 in cHEK293 cells that transiently overexpress hGLUT‐1 to ‐4, respectively, showed that most compounds are unselective among the four GLUT isoforms (Table 2). Compound **155** reduced lactate excretion after 4 h treatment with an IC_50_ value of about 90 nm (5 mm glucose) and 400 nm (17 mm glucose)^19^ and induced apoptosis in A459 cells with an IC_50_ value of 0.126 μm as determined by means of live‐cell imaging using a nuclear stain for cell counting (Table 2).^19^ To date, biological applications of these GLUT inhibitors have not been reported.

Kapoor et al. reported peptide analogues GLUT‐i1 and GLUT‐i2 (Figure 2), with glucose uptake inhibitory activity. The cellular assay that was used monitored ATP depletion of hGLUT‐1‐ and luciferase‐transfected CHO‐K1 cells in the presence of a mitochondrial complex I inhibitor.^20^ GLUT‐i1 and GLUT‐i2 inhibit glycolytic ATP production with IC_50_ values of 267 and 140 nm, respectively (Table 1).^20^ GLUT isoform selectivity was determined in the same assay using DLD‐1 cells (express mainly GLUT‐1) and DLD‐1 *GLUT1* (−/−) cells (express mainly GLUT‐3) or CHO cells that were stably transfected with hGLUT‐2 or hGLUT‐4. GLUT‐i1 and GLUT‐i2 preferably inhibit GLUT‐1 and GLUT‐4 over GLUT‐2 and GLUT‐3 (Table 2).^20^ GLUT‐i1 competes with glucose for the same binding site as determined in glucose competition experiments and crystal structure analysis with hGLUT‐1 (Table 2).^20^ However, an impact of GLUT‐i1 or GLUT‐i2 on cancer cell growth has not been described.

Bayer researchers developed 1*H*‐pyrazolo[3,4‐*d*]pyrimidine GLUT inhibitors.^21^ Compound **3** (patent: example 1) inhibits glucose uptake with an IC_50_ value of 25 nm in rotenone‐treated DLD‐1 cells in an assay monitoring ATP production. It competitively inhibits glucose uptake by targeting GLUT‐1 and GLUT‐3 without interfering with GLUT‐2 activity. Siebeneicher et al. further characterized compound **3** for its in vitro and in vivo pharmacokinetics.21b

In addition, Bayer reported the first highly GLUT‐1‐selective compound based on the *N*‐(1*H*‐pyrazolo‐4‐yl)quinoline‐4‐carboxamide scaffold.^22^ BAY‐876 (IC_50_=2 nm, Figure 2, Table 1) possesses a clear preference for GLUT‐1 over GLUT‐2, ‐3, and ‐4. It competitively inhibits GLUT‐1 (Table 2) and shows good in vitro and in vivo properties.22a However, BAY‐876 did not inhibit the growth of the triple‐negative breast cancer cell lines BT549, MDA‐MB‐436, and HCC70 at 3 μm as determined by live‐cell imaging (Table 2).^23^ Ma et al. applied BAY‐876 to different ovarian cancer cell lines using an MTT assay and determined a growth inhibitory IC_50_ value of 60 nm for OVCAR‐3 cells (Table 2), whereas SKOV‐3 cells yielded an IC_50_ value of 188 nm and A2780 cells were resistant to treatment.^24^ Hence, the potency of BAY‐876 seems to be cell line dependent. Furthermore, daily treatment with 4.5 mg kg^−1^ BAY‐876 over 2 weeks decreased the tumor volume of SKOV‐3 xenograft models by 68 % (Table 2). Despite a loss of body weight, the mice showed no major health impairment.^24^ BAY‐876 selectively inhibits GLUT‐1 in vitro and in vivo. However, care should be taken when selecting an appropriate cell line. BAY‐876 has been successfully applied to esophageal squamous cancer cell lines TE‐8 and TE‐11 in combination with cisplatin.^25^

Kadmon reported a different class of glucose uptake inhibitors.^26^ Example **31** in the corresponding patent (Figure 2) inhibited glycolytic ATP generation in HT1080 cells that were treated with oligomycin (inhibitor of mitochondrial complex V; Table 1). It potently inhibits the proliferation of Jurkat (294 nm), MOLT‐4 (385 nm), and U937 cells (256 nm) as measured after 72 h in a sulforhodamine B assay (Table 2).^26^

Chromopynone‐1 (Figure 2) was identified by Waldmann et al. as a potent GLUT‐1/‐3 isoform‐selective inhibitor. Chromopynone‐1 was developed from a chromane–tetrahydropyrimidone hit class that was identified in a cell‐based screen monitoring the uptake of 2DG by enzyme‐coupled resazurin detection in HCT116 cells.^27^ It inhibits glucose uptake (IC_50_=414 nm) in a GLUT‐1‐/‐3‐selective manner as determined with partial rescue of 2DG uptake inhibition in CHO cells that transiently overexpress hGLUT‐1 to ‐4 (Tables 1 and 2). Growth of HCT116 cells was inhibited by chromopynone‐1 with a GI_50_ value of >25 μm (25 mm glucose) and 3.8 μm (5 mm glucose) and of MIA PaCa‐2 cells with a GI_50_ value of 2.8 μm (25 mm glucose) and 0.6 μm (5 mm glucose) as determined by live‐cell imaging (Table 2). MIA PaCa‐2 cells depend more on glucose than HCT116 cells.^28^

Waldmann et al. also discovered piperazin‐2‐one‐derived inhibitors in the same cell‐based assay. The most active member, glutor (IC_50_=11 nm, Figure 2, Table 1), targets GLUT‐1, ‐2 and ‐3 (Table 2).^27^, ^29^ Cell line sensitivity was tested using 94 different cell lines using a sulforhodamine B assay for 72 h of glutor treatment, and revealed that the nonmalignant cell line IMR‐90 and peripheral blood mononucleated cells (PBMCs) were resistant (IC_50_>30 μm) to treatment with glutor, whereas nearly half of the malignant cell lines exhibited IC_50_<100 nm (Table 2).^29^ The urinary bladder carcinoma cell line UM‐UC‐3 and MIA PaCa‐2 cells were most sensitive (IC_50_=4 nm, Table 2).^29^ The fact that some cancer cell lines were resistant to glutor treatment indicates different susceptibilities, as observed for BAY‐876, which was traced back to the metabolic phenotype of the cells and their potential to switch flexibly between a glycolytic and an oxidative phenotype.^29^ Furthermore, glutor decreased glycolytic flux, lactate excretion and viability of HCT116 cells to a similar extent in monolayer cultures and in spheroids which indicates potential applicability in animal models (Table 2).^29^

In addition, Waldmann et al. described indomorphan GLUT‐inhibitors. The most active derivative glupin (IC_50_=4 nm, Figure 2, Table 1) is a mixed‐type inhibitor that targets GLUT‐1 and GLUT‐3 (Table 2).^30^ Furthermore, glupin decreases the glycolytic flux in MDA‐MB‐231 cells which was confirmed in metabolomic measurements monitoring glycolytic metabolites in MOLT16 cells.^30^ The growth of various malignant cell lines was suppressed by glupin (IC_50_(UM‐UC‐3)=31 nm; IC_50_(MIA PaCa‐2)=62 nm, Table 2) which was monitored using a sulforhodamine B assay.^30^ PBMCs as well as the nonmalignant cell line IMR‐90 were resistant to glupin treatment (IC_50_>30, Table 2).^30^

NV‐5440 was recently discovered by Kang et al. while searching for mTORC1‐selective compounds in a cell‐based high‐throughput screen (Figure 2).^31^ Target identification approaches using a SILAC (stable isotope labeling by amino acids in cell culture) assay revealed that GLUT‐1 was the major target of NV‐5440 which could be further confirmed by metabolic flux analysis of downstream metabolites within the glycolytic cascade.^31^ NV‐5440 inhibited the uptake of [^3^H]2DG with an IC_50_ value of 36 nm (Table 1) in a GLUT‐1‐ to ‐4‐dependent manner while leaving GLUT‐5 unaffected (Table 2). Selectivity was determined with stably transfected CHO‐K1 (hGLUT‐1 to ‐3) or HEK293‐T (hGLUT‐4 to ‐5) cells using an ATP depletion assay in the presence of rotenone. Control experiments revealed that glucose depletion is responsible for the selective mTORC1 inhibition, which could be confirmed in vivo.^31^ Moreover, in vivo pharmacokinetic data are available for NV‐5440 and the metabolically more stable derivative NV‐6297 (Figure 2).^31^

## 3 GLUT Isoform Selectivity Profiles to Target Cancer

GLUTs exhibit a tissue‐specific distribution and selective expression in cancer.^8^, ^32^ GLUT‐1 is expressed at high levels in most cancers, and GLUT‐3 is predominantly found in the brain, arguing for the development of GLUT‐1‐selective compounds.22a However, GLUT‐3 is also overexpressed in numerous additional cancer types (beyond glioblastoma that originates from nervous tissue32b) such as breast and endometrial cancer, head and neck tumors, colon cancer, pancreatic cancer, non‐small cell lung cancer and thyroid carcinomas.^33^ Vander Heiden proposed that targeting GLUT‐3 might enlarge the therapeutic window of glucose uptake inhibitors, as GLUT‐3 is only expressed in a small fraction of somatic cells (mainly neurons), but is overexpressed in many cancers.^6^

BAY‐876 is selective for GLUT‐1,22a but it is unclear if the GLUT‐1 isoform selectivity is necessary or sufficient. Chromopynone‐1, glutor and glupin target the isoforms GLUT‐1 and GLUT‐3 and DLD‐1 cells upregulate GLUT‐1 and GLUT‐3 after 24 h and 48 h when cultured under hypoglycemic conditions.^29^, ^30^ This adaptation mechanism was mimicked by the treatment with 0.5 μm glutor and 0.5 μm glupin, respectively.^29^, ^30^ *GLUT4* mRNA stayed unaltered and *GLUT2* mRNA was not detectable in these cells, indicating a low relevance for this isoform under hypoglycemic conditions.^29^, ^30^ Similar outcomes were observed previously in glucose‐deprived neuronal rat cells and in MCF7 and HeLa cells cultured under reduced (2.5 mm) glucose concentration.^34^ The increased expression of GLUT‐3 might be a natural rescue mechanism of neuronal cells to ensure glucose uptake in a hypoglycemic environment. Because GLUT‐3 has the highest affinity for glucose among the GLUT isoforms (*K*_M_(2DG)=1.4 mm),^35^ cells expressing GLUT‐3 have an advantage in competing for glucose with the surrounding tissue. Hence, a GLUT‐1‐/‐3‐selective inhibitor may be necessary in order to completely block the glucose uptake of cancer cells.

However, this observation might be cell line specific, because a reduced glucose level (2 mm glucose) or the treatment of A549 cells with WZB117 increased *GLUT1* mRNA after 24 h, but decreased GLUT‐1 protein levels after 12, 24, and 48 h.17b The authors explained these results by a rapid upregulation of *GLUT1* mRNA under low glucose conditions, but restricted energy and glucose supply cannot fuel the biosynthesis of the glycosylated GLUT‐1 protein.17b Also thyroid cancer cell lines (FTC‐133 and 8305c) increased GLUT‐1 protein expression after 48 h incubation with 5 and 2 mm glucose, compared with 25 mm glucose. GLUT‐3 protein levels stayed unaltered, confirming a cell‐dependent effect.^36^

## 4 Combination Studies

Many cancers show high metabolic plasticity, because mitochondria are usually still functional and can use alternative nutrients for energy production and biosynthesis.^37^ To explore synergistic targeting of several metabolic pathways, the glucose uptake inhibitor glutor was combined with CB‐839,^38^ a small‐molecule inhibitor that targets the kidney glutaminase isoform which is overexpressed in many cancers, to suppress the growth of HCT116 cells.^29^ The combination of glutor with CB‐839 decreased the GI_50_ value of glutor from 428 nm (0 μm CB‐839) by about 40‐fold to GI_50_=10 nm (5 μm CB‐839).^29^ Inhibition of the glutaminase disrupts the supply of α‐ketoglutarate to the TCA cycle and therefore interferes with an alternative metabolic pathway for energy production. The availability of the amino acid aspartate has also a strong impact on cell survival, as it influences the dependence of cell on glutamine and could hence offer another approach for a combinatory treatment.^39^

Combining chemotherapeutic agents with glucose uptake inhibitors has already led to promising results. A reason could be that most chemotherapeutic agents elevate reactive oxygen species (ROS) levels and thereby influence redox status of the cancer cell.^40^ Treatment of MCF‐7 breast cancer cells with WZB117 partially restored sensitivity of the cells toward the chemotherapeutic agent adriamycin.18j WZB117 has also been successfully applied together with 5‐fluorouracil on resistant colon carcinomas (HCT116), which can be most probably explained with an observed GLUT‐1 upregulation in 5‐fluorouracil‐resistant and ‐treated colon cells.18p The GLUT‐1‐selective inhibitor BAY‐876 enhanced the response of cisplatin‐treated esophageal squamous cell carcinoma with respect to cell proliferation.^25^ Radiation of a tumor acts through creating double‐strand breaks in DNA as well as through cellular water radiolysis, which creates ROS.^41^ Hence, a combinatorial treatment of radiotherapy and glucose uptake inhibition might offer a promising opportunity to target cancer more efficiently. An increase in GLUT‐1 expression and higher glycolytic activity was observed upon radiotherapy treatment and in radiotherapy‐resistant breast cancer cells.18l The authors observed that simultaneous treatment of breast cancer cells with WZB117 sensitized the resistant cells to radiotherapy.18l The simultaneous treatment of hepatocellular carcinoma with 2DG and with kinase inhibitor sorafenib also showed promising results in targeting sorafenib‐resistant populations in vitro and in vivo.^42^ Overall, the inhibition of glycolysis, for example, by glucose transporter inhibitors, seems to be highly effective to sensitize cancer to diverse treatment approaches.

## 5 Possible Applications beyond Oncology

**Figure 3:**
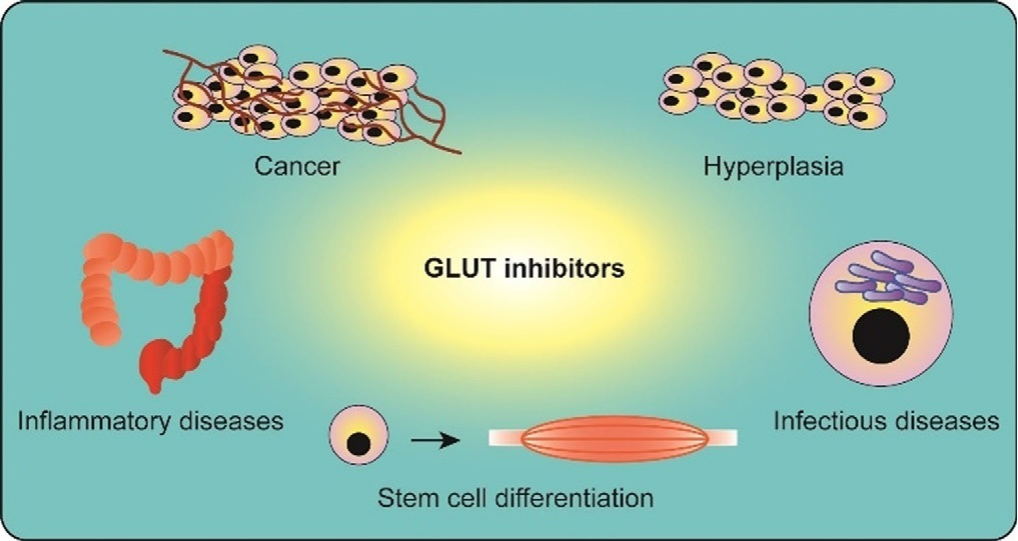
Overview of potential therapeutic applications of GLUT inhibitors.

Aerobic glycolysis and increased glucose dependence are also characteristic for inflammatory diseases (Figure 3). CD4^+^ T cells switch from fatty acid β oxidation in the resting state to aerobic glycolysis after activation. Interestingly, GLUT‐1‐deficient CD4^+^ T cells were unable to grow, proliferate, survive and differentiate to T effector cells after activation.2a T cells that upregulate aerobic glycolysis are involved in the establishment of inflammatory bowel disease, graft‐versus‐host disease and systemic lupus erythematosus.^2^, ^43^ Notably, in systemic lupus, autoreactive CD4^+^ T cells upregulate oxidative phosphorylation along with glycolysis, and combinatorial treatment with 2DG and metformin showed promising results in mouse models.2b Also, HIV‐infected patients hold a large number of CD4^+^ T cells, which overexpress GLUT‐1.^44^

Hyperplasia‐associated diseases, such as psoriasis and fibrosis, exhibit uncontrolled cell proliferation and increased GLUT‐1 levels, offering potential for modulation by treatment with glucose import inhibitors (Figure 3).^3^, ^45^ Age‐related macular degeneration (AMD) is characterized by ocular neovascularization. Because increased levels of glycolysis have been observed in endothelial cells and AMD patients exhibit increased lactate/pyruvate ratios, restriction of glycolysis might be a promising therapeutic approach to interfere with the endothelial proliferation.^46^

Intracellular bacteria and parasites may manipulate the host cell's metabolism to increase glycolysis (Figure 3). The bacteria *Chlamydia trachomatis*,^47^ *Chlamydia pneumoniae*,^48^ *Mycobacterium tuberculosis*,^49^ *Brucella abortus* 50 and *Legionella pneumophila* 51 have been reported to induce a Warburg‐like phenotype of their host cells. Treatment with 2DG reduced the replication of *L. pneumophila* inside human macrophages.^51^, ^52^ The malaria parasite *Plasmodium falciparum* replicates inside erythrocytes and increases GLUT‐1 expression of the host cells in order to fuel its own metabolism.^53^ Recently, Wei et al. successfully applied WZB117 to plasmodium‐infected erythrocytes, which induced oxidative stress and apoptosis.18e Viral infections also lead to an adaptation of the energy metabolism of the host cells toward aerobic glycolysis. Cells infected by rhinovirus increase GLUT‐1 expression and release additional glucose from their glycogen storage. Treatment with glycolysis inhibitor 2DG reverts the metabolism to lipogenesis. Thus GLUT inhibitors might open an alternative opportunity to address viral infection.^54^

Furthermore, the transformation of progenitor cells to differentiated cells often involves a switch in the metabolic phenotype of the cell (Figure 3). Izumi et al. recently demonstrated that the treatment of connective tissue with 2DG drives the differentiation to tendon cells and inhibits chondrogenesis, which is associated with poor tendon healing.^55^ Applying small molecules in the field of directed differentiation might offer tremendous potential.

## 6 Summary and Outlook

Aerobic glycolytic phenotypes have been observed in multiple diseases. Targeting altered glucose uptake and metabolism with appropriate tool compounds, such as GLUT inhibitors, could yield new insight in the diseases and pave the way for novel therapeutic strategies. Eleven distinct compound classes that inhibit glucose uptake with sub‐micromolar potency and target the glucose transporters with different GLUT isoform selectivity have been reported. These compounds have been further characterized biologically and provide a valuable tool compound platform to further investigate glucose metabolism in different disease models.

For less‐well‐characterized GLUT isoforms of class II and III, selective tool compounds are not yet available. As these isoforms are also overexpressed in some cancers and other diseases,^56^ this field of research could offer additional opportunities for the treatment of disease.

## Conflict of interest

*H.W. is sponsor of a drug discovery program at the Lead Discovery Center of the Max‐Planck‐Gesellschaft aimed at the development of GLUT inhibitors*.

## Biographical Information

Elena S. Reckzeh (née Heider) obtained her Bachelor′s degree in molecular biomedicine at the University of Bonn and her Master′s degree in chemical biology at the TU Dortmund University in 2014. In 2019, she completed her PhD in chemical biology under the guidance of Herbert Waldmann at the Max Planck Institute (MPI) of Molecular Physiology in Dortmund and worked on the synthesis and biological evaluation of novel glucose transporter inhibitors.

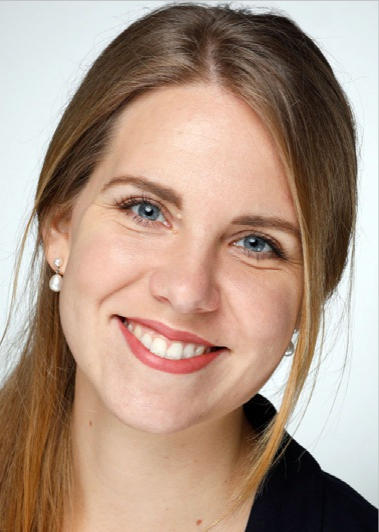

## Biographical Information

Herbert Waldmann obtained his PhD in organic chemistry in 1985 under the supervision of Horst Kunz. After a postdoctoral period with George Whitesides at Harvard University, he returned to the University of Mainz and completed his habilitation in 1991. He was appointed as director at the MPI Dortmund and professor of Organic Chemistry at TU Dortmund University in 1999. His research focuses on the syntheses of natural‐product‐derived compounds and their biological evaluation.